# Supplementary material for: Associations of Maternal Urinary Concentrations of Phenols, Individually and as a Mixture, with Serum Biomarkers of Thyroid Function and Autoimmunity: Results from the EARTH Study
Source: Toxics. 2023 Jun 9;11(6):521. doi: 10.3390/toxics11060521 (PMC10302981; doi:10.3390/toxics11060521)
Supplement: Supplementary file 1 [file toxics-11-00521-s001.zip › toxics-2378259-supplementary.pdf]

Table S1. Distribution of urinary concentrations (µg/L) of phenol biomarkers among women in the Environment and Reproductive (EARTH) Study.

	Detection Frequency[a] %	Geomean (SE)	10th	25th	50th	75th	95th
Bisphenol A	85	0.86 (0.06)	0.28	0.30	0.80	1.80	5.80
Benzophenone-3	99	93.6 (10.6)	7.20	23.3	84.6	377	3090
Butylparaben[b]	57	0.53 (0.06)	0.07	0.14	0.20	2.60	24.9
Methylparaben	100	80.2 (7.10)	7.80	30.4	85.7	249	1080
Propylparaben[b]	98	16.5 (1.80)	0.80	4.30	23.1	66.8	293
Triclosan	79	11.7 (1.31)	1.63	1.70	7.10	47.2	488

[a]Limits of detection (LOD) ranged from 0.1 to 1.0 µg/L. Note: Data are presented before imputation of concentrations <LOD.

[b]Not considered in further analyses.

Table S2. Linear single-exposure model results in EARTH Study. Each column/row pair corresponds to a separate model fit.

	log-bisphenol A		log-benzophenone-3		log-methylparaben		log-triclosan	
	**Est**	**95% CI**	**Est**	**95% CI**	**Est**	**95% CI**	**Est**	**95% CI**
**TSH**	0.113	(0.051, 0.175)	-0.008	(-0.039, 0.023)	0.043	(0.001, 0.084)	-0.032	(-0.058, -0.005)
**fT_4**	-0.141	(-0.259, -0.023)	-0.047	(-0.104, 0.011)	0.143	(0.066, 0.220)	-0.013	(-0.063, 0.036)
**TT_4**	-2.201	(-3.330, -1.073)	-0.243	(-0.803, 0.318)	-0.218	(-0.97, 0.533)	0.095	(-0.381, 0.571)
**fT_3**	-0.081	(-0.113, -0.048)	-0.011	(-0.027, 0.005)	0.035	(0.013, 0.056)	-0.039	(-0.052, -0.025)
**TT_3**	-0.062	(-0.085, -0.039)	0.005	(-0.006, 0.017)	0.010	(-0.005, 0.026)	-0.011	(-0.021, -0.001)
**TgAb**	1.015	(0.845, 1.219)	0.950	(0.865, 1.044)	1.243	(1.090, 1.418)	0.893	(0.822, 0.970)
**TPOAb**	1.113	(0.932, 1.328)	0.924	(0.843, 1.013)	1.010	(0.893, 1.143)	0.943	(0.869, 1.022)

Note: Est is estimated association for each 1 unit increase in log concentrations; 95% CI is corresponding confidence interval. For binary outcomes TgAb and TPOAb, coefficients represent odds ratios; all others are mean differences. Models were adjusted for age (years), BMI (kg/m2), race (white vs. other), and specific gravity (SG)..

Table S3. Linear mixture model results in EARTH Study. Each row corresponds to a single model fit.

	log-bisphenol A		log-benzophenone-3		log-methylparaben		log-triclosan	
	**Est**	**95% CI**	**Est**	**95% CI**	**Est**	**95% CI**	**Est**	**95% CI**
**TSH**	0.109	(-0.013, 0.231)	-0.016	(-0.078, 0.046)	0.043	(-0.040, 0.125)	-0.030	(-0.082, 0.023)
**fT_4**	-0.150	(-0.382, 0.083)	-0.060	(-0.175, 0.055)	0.166	(0.012, 0.319)	-0.015	(-0.111, 0.081)
**TT_4**	-2.160	(-4.400, 0.079)	-0.134	(-1.258, 0.990)	-0.080	(-1.577, 1.417)	0.069	(-0.867, 1.005)
**fT_3**	-0.088	(-0.151, -0.025)	-0.008	(-0.040, 0.024)	0.043	(0.010, 0.086)	-0.041	(-0.067, -0.014)
**TT_3**	-0.066	(-0.112, -0.020)	0.008	(-0.015, 0.032)	0.012	(-0.019, 0.043)	-0.013	(-0.033, 0.006)
**TgAb**	1.004	(0.697, 1.447)	0.943	(0.781, 1.139)	1.265	(0.975, 1.641)	0.892	(0.757, 1.051)
**TPOAb**	1.123	(0.793, 1.590)	0.919	(0.764, 1.106)	1.027	(0.806, 1.310)	0.949	(0.808, 1.115)

Note: Est is estimated association for each 1 log unit increase in concentrations; 95% CI is corresponding confidence interval. For binary outcomes TgAb and TPOAb, coefficients represent odds ratios; all others are mean differences. Models were adjusted for age (years), BMI (kg/m2), race (white vs. other), and specific gravity (SG).

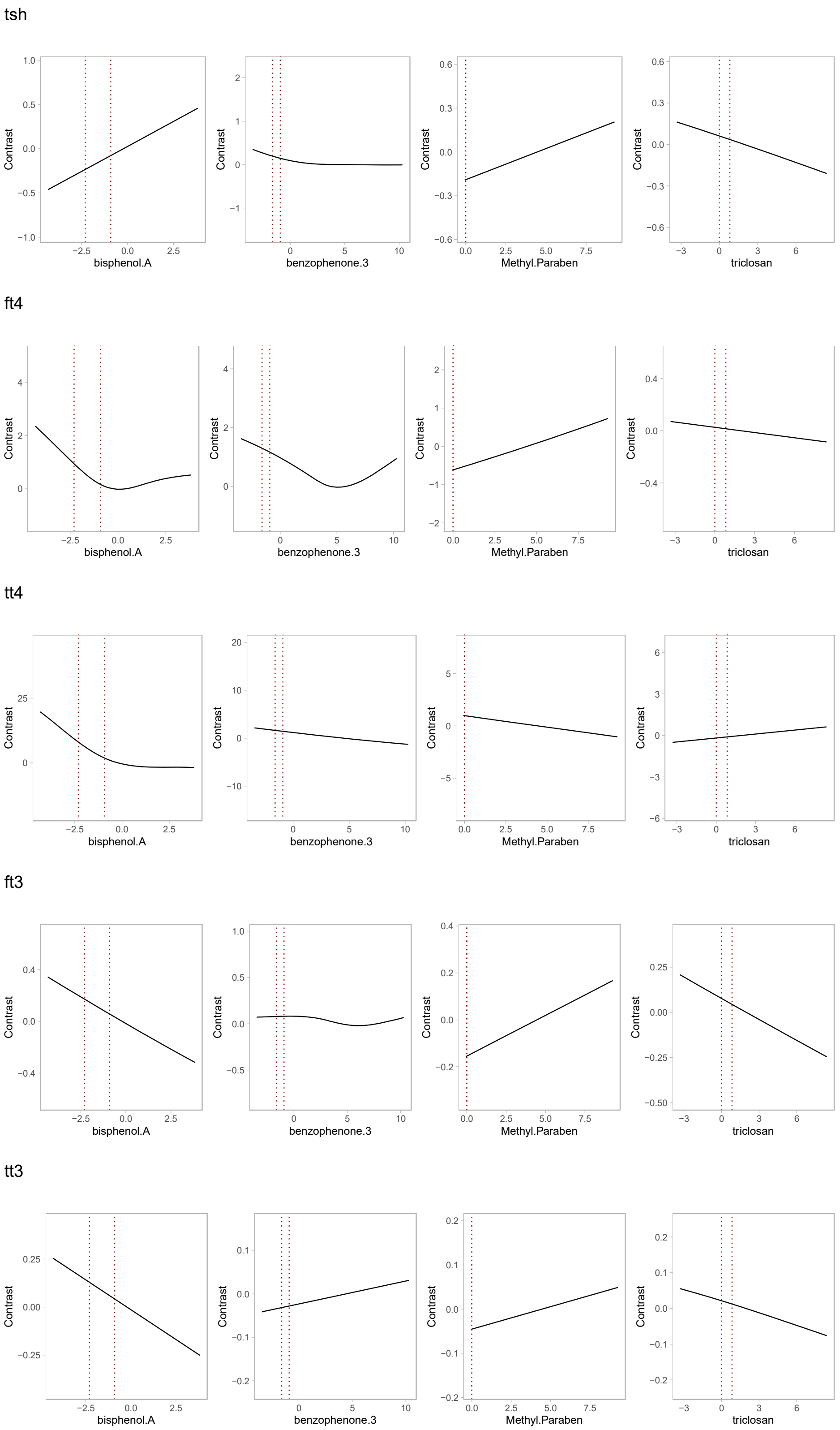

Figure S1. Single-component analysis: additive model results for continuous outcomes. Curves are estimated mean differences and corresponding 95% confidence intervals, compared to median log concentration. X-axis indicates log concentration. Each plot corresponds to an individual model. Models were adjusted for age (years), BMI (kg/m2), race (white vs. other), and specific gravity (SG).

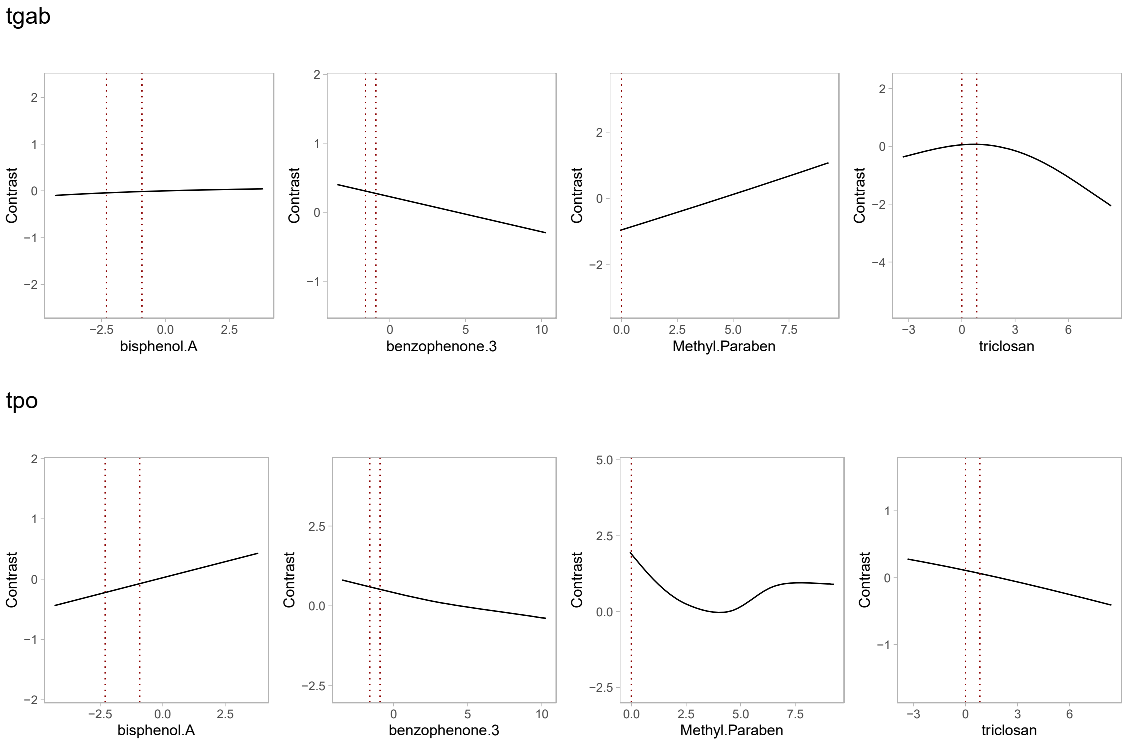

Figure S2. Single-component analysis: generalized additive model results for binary outcomes. Curves are estimated log odds ratios and corresponding 95% confidence intervals, compared to median log concentration. X-axis indicates log concentration. Each plot corresponds to an individual model. Models were adjusted for age (years), BMI (kg/m2), race (white vs. other), and specific gravity (SG).

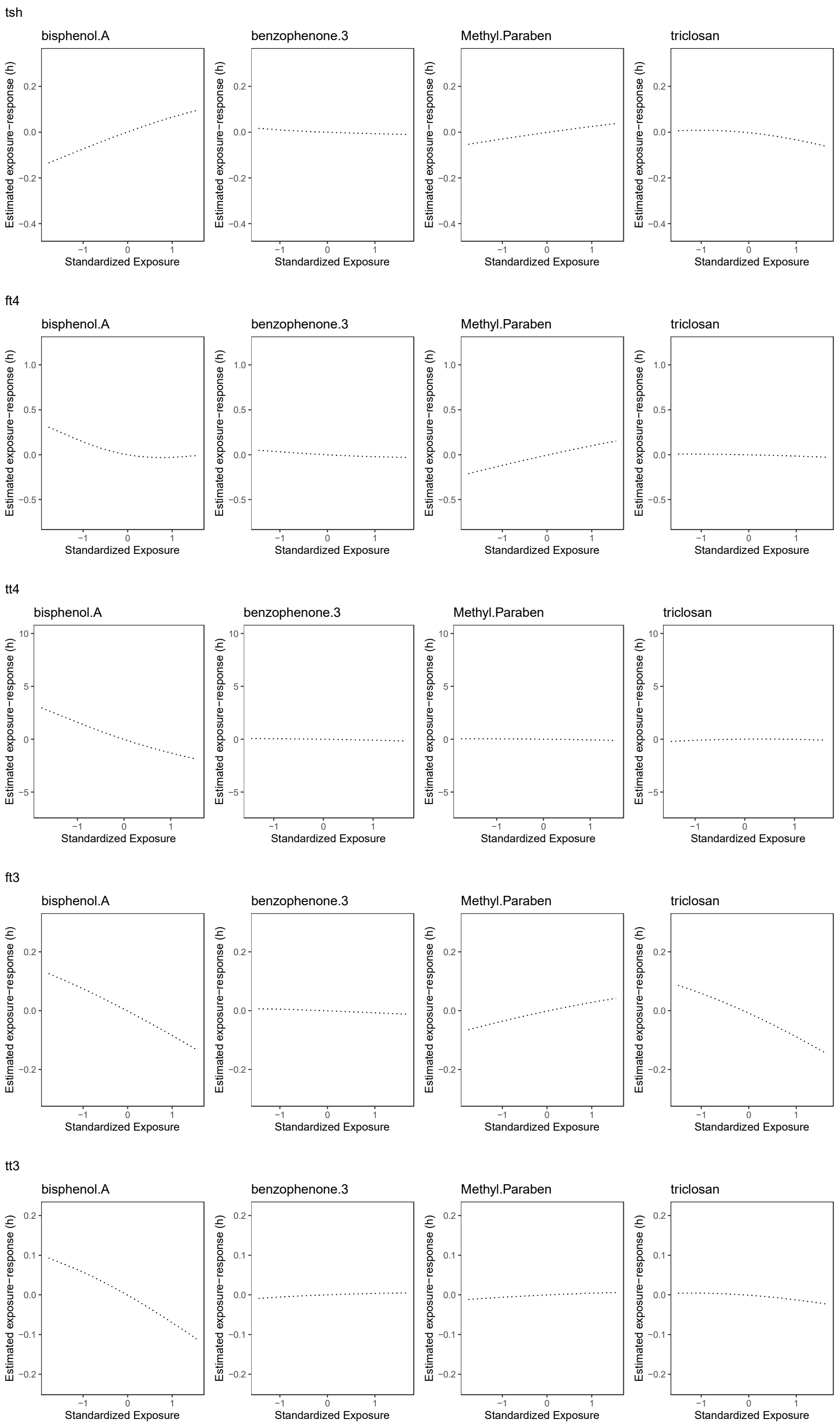

Figure S3. Mixture analysis: BKMR results for continuous outcomes. Curves are estimated mean differences and corresponding 95% credible intervals. Each row corresponds to a different model. Models were adjusted for age (years), BMI (kg/m2), race (white vs. other), and specific gravity (SG).

(a)

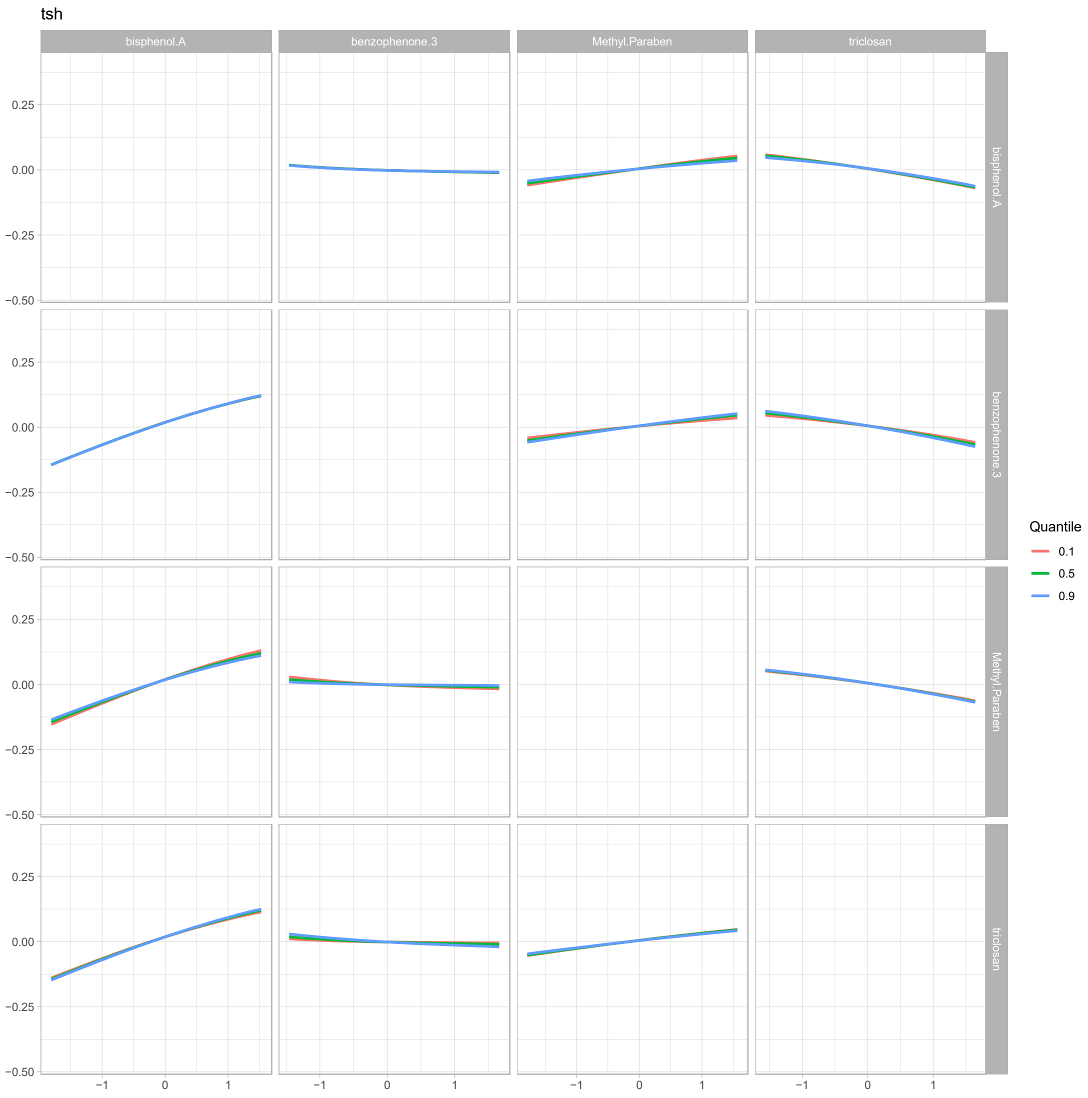
tsh
bisphenol.A
benzophenone.3
Methyl.Paraben
triclosan
0.25
0.00
−0.25
−0.50
−1
0
1
Quantile
0.1
0.5
0.9

(b)

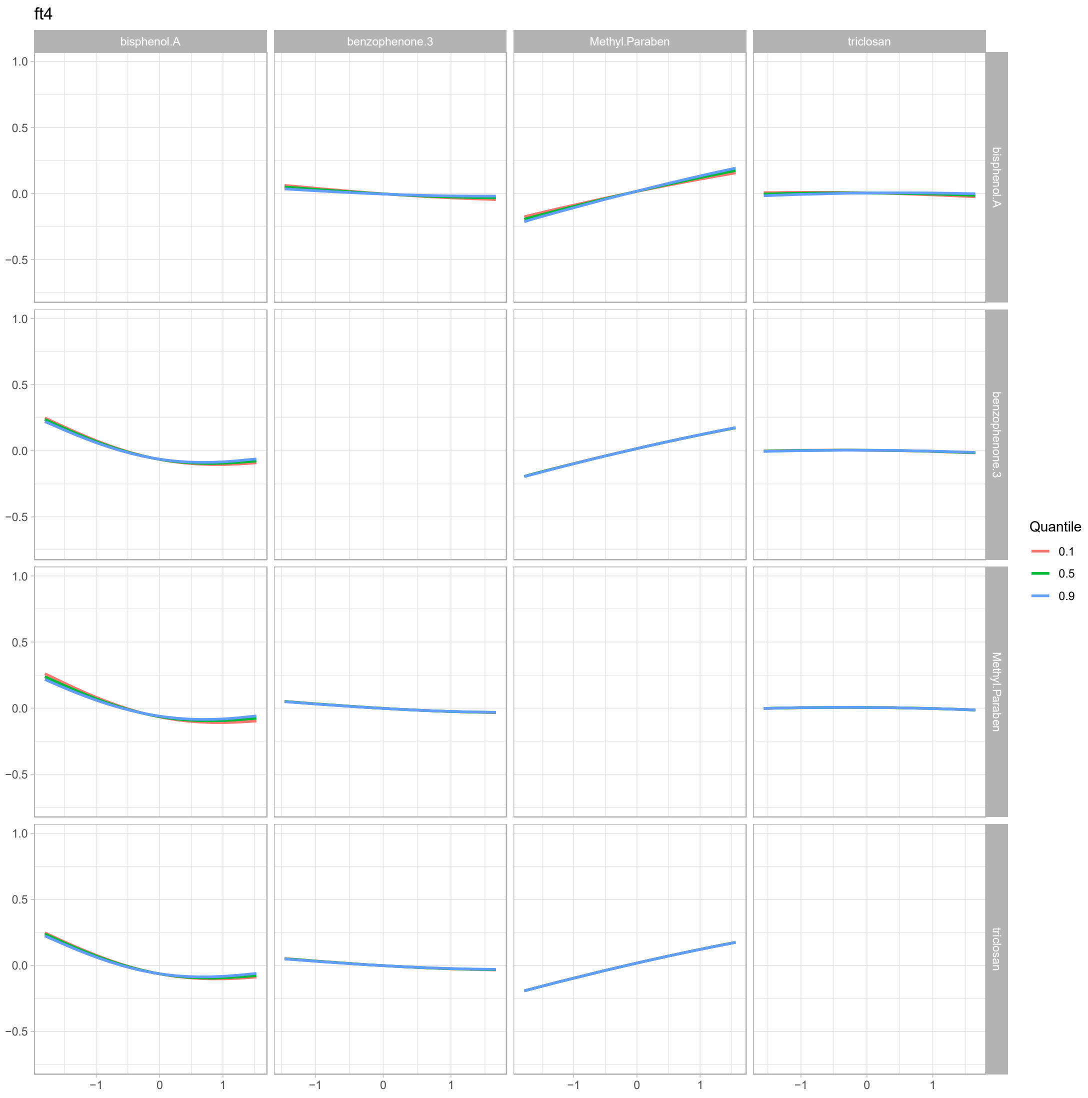
ft4
bisphenol.A
benzophenone.3
Methyl.Paraben
triclosan
Quantile
0.1
0.5
0.9
1.0
0.5
0.0
−0.5
−1
0
1

(c)

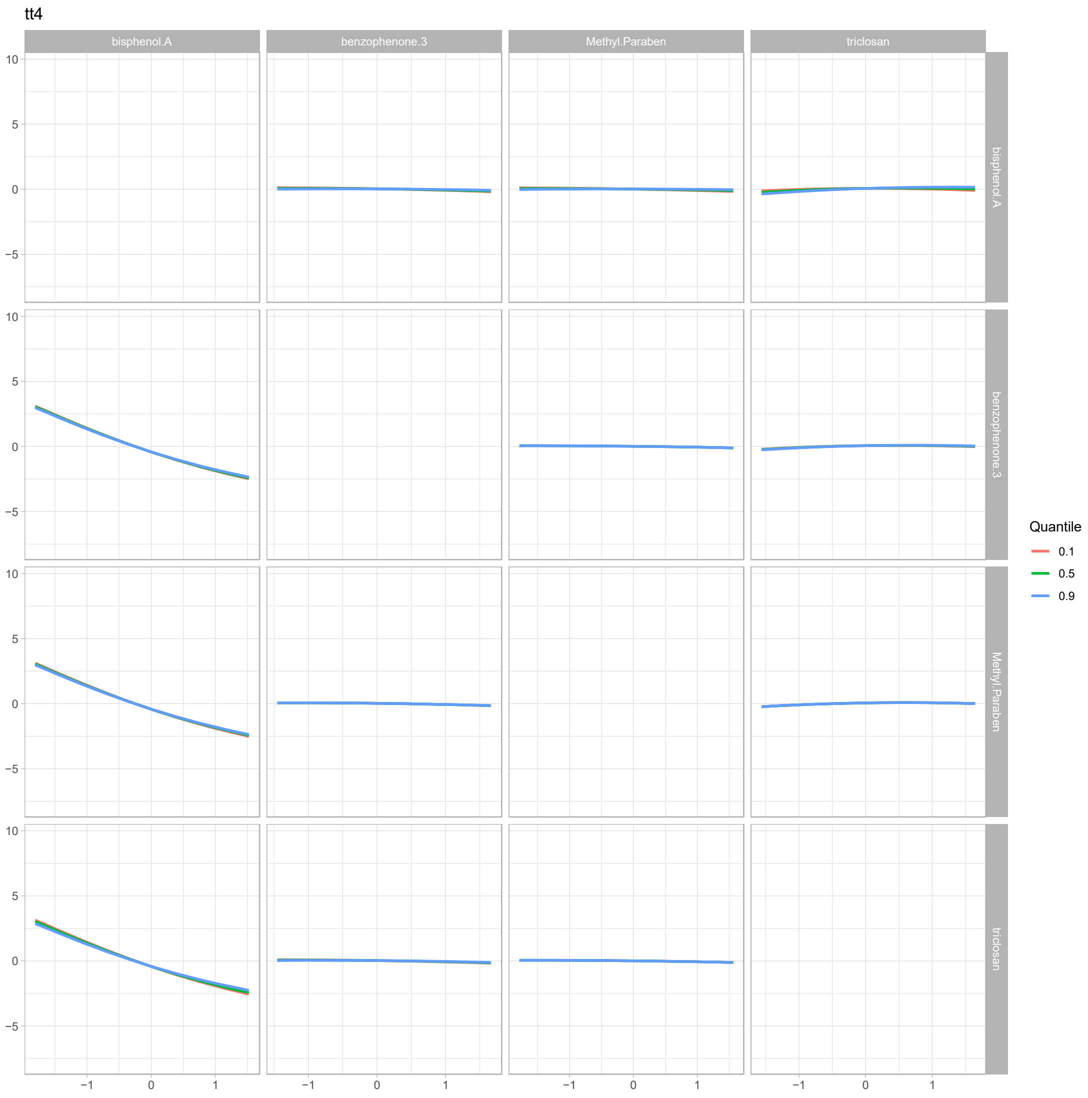
tt4
bisphenol.A
benzophenone.3
Methyl.Paraben
triclosan
Quantile
0.1
0.5
0.9
10
5
0
−5
−1
0
1

(d)

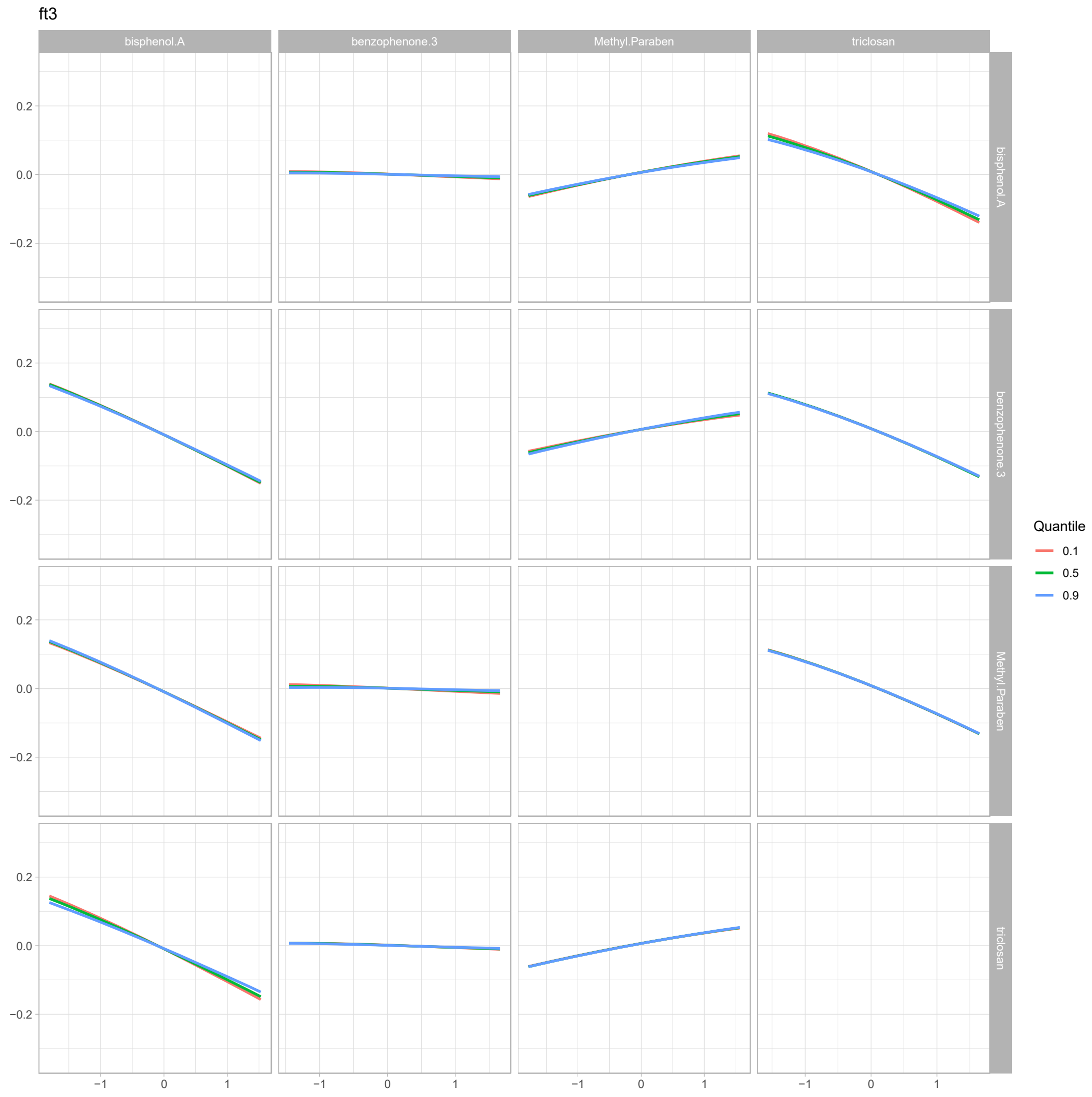
ft3
bisphenol.A
benzophenone.3
Methyl.Paraben
triclosan
0.2
0.0
−0.2
−1
0
1
Quantile
0.1
0.5
0.9

(e)

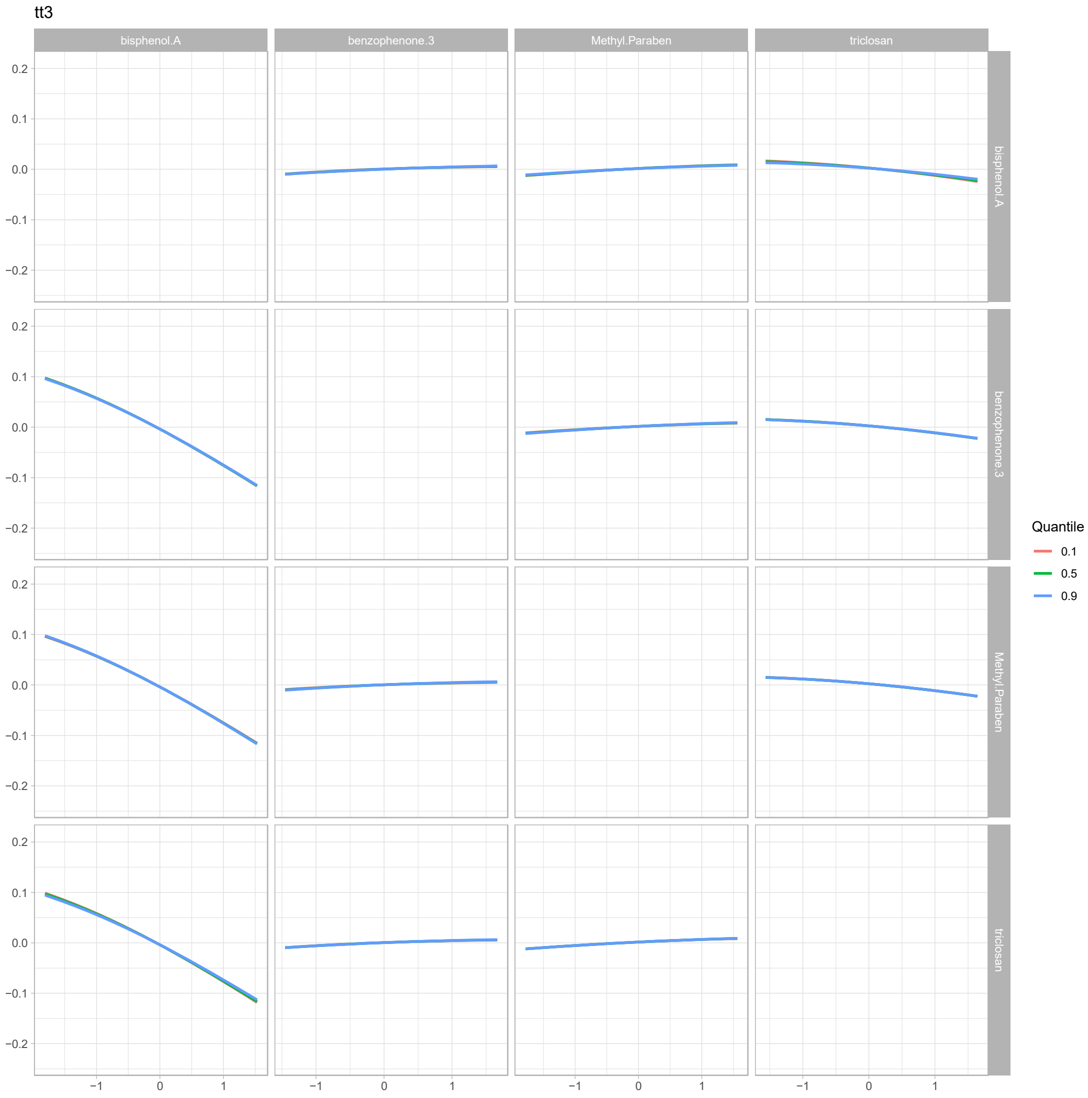

Figure S4. Mixture analysis: pairwise interaction plots for in the BKMR analyses for (a) TSH, (b) fT_4, (c) fT_3, (d) TT_4 and (e) TT_3. Curves are estimated mean differences and corresponding 95% credible intervals, holding y-axis component at 10th, 50th or 90th percentile, and other components set to their median. Models were adjusted for age (years), BMI (kg/m2), race (white vs. other), and specific gravity (SG).
